# Characterization of regional meniscal cell and chondrocyte phenotypes and chondrogenic differentiation with histological analysis in osteoarthritic donor-matched tissues

**DOI:** 10.1038/s41598-020-78757-6

**Published:** 2020-12-10

**Authors:** Jingsong Wang, Sally Roberts, Jan Herman Kuiper, Weiguo Zhang, John Garcia, Zhanfeng Cui, Karina Wright

**Affiliations:** 1 School of Pharmacy and Bioengineering, Keele University, Keele, ST5 5GB Staffordshire UK; 2 The Robert Jones and Agnes Hunt Orthopaedic Hospital NHS Foundation Trust, Oswestry, SY10 7AG Shropshire UK; 3 Dalian Medical University, Dalian, 116044 China; 4 Department of Orthopaedic Surgery, First Affiliated Hospital, Dalian Medical University, Dalian, 116011 China; 5 Department of Engineering Science, Institute of Biomedical Engineering, University of Oxford, Oxford, OX1 3PJ UK

**Keywords:** Cell biology, Medical research

## Abstract

Meniscus degeneration is closely related to the progression of knee osteoarthritis (OA). However, there is currently a lack of quantitative and objective metrics to assess OA meniscal cell phenotypes. In this study we investigated the phenotypic markers and chondrogenic potency of avascular and vascular meniscal cells and chondrocytes from medial OA knee joints (n = 10). Flow cytometry results showed that a significantly greater percentage of meniscal cells were positive for CD49b, CD49c and CD166 compared to donor-matched chondrocytes after 14 days in monolayer culture. The integrins, CD49b and CD29, were expressed at a significantly higher level on avascular meniscal cells derived from tissues with a more degenerated inner border than non-degenerate menisci, suggesting that the integrin family may play an important role in meniscus OA pathology. Collagen fibres arranged in a “tree-like” formation within the meniscus appeared to have less blood vessels associated with them in the vascular region of the most degenerate menisci, which may indicate that such structures are involved in the pathological process. We have demonstrated that meniscal cells derived from the lateral meniscus in medial OA patients have chondrogenic capacity in vitro and hence could represent a potential cell source to consider for meniscus tissue engineering.

## Introduction

An increasing number of studies suggest that meniscal degeneration plays a significant role in the pathology of osteoarthritis (OA)^1^. Certainly meniscal degeneration is a classical feature of OA knee joints, as seen on Magnetic Resonance Imaging (MRI), contributing to a substantial proportion of joint space narrowing observations^2^. In addition, degenerative tears of the meniscus have important consequences for cartilage loss, as the tears interfere with converting axial loading into horizontal tensile strain and subsequently increasing contact stress on the articular cartilage^3^. Together, this evidence suggests that pathological changes and extracellular matrix (ECM) degeneration in menisci may play an important role in the disease process of OA.

A series of histological features of the degenerate meniscus were reported in a previous study^4^, including fibrocartilaginous separation of the matrix, fraying, tears, calcification, diffuse hypercellularity, cellular hypertrophy and the presence of abnormal cell clusters within the meniscus matrix. A recent study also demonstrated the severe disorganization of collagen fibers and increased proteoglycan by electron microscopy and histology in late-stage OA patients^5^. In addition, in another study the biochemical composition of the OA meniscus was shown to be altered, in terms of extracellular matrix disorganization, disturbances in collagen and non-collagen protein synthesis and gene expression^6^. However, the mechanism(s) behind these degenerative changes is still unclear. Investigating the surface marker and gene expression profiles of meniscal cells from OA knees could contribute to this knowledge base.

The meniscus contains a heterogeneous cell population; at least three cell fractions are generally accepted to reside within the tissue^7^. In the inner and middle part of the meniscus (the avascular zone), the main cell type has been defined as having a round or oval shape and the cells have been termed fibrochondrocytes^8^. The outer one-third proportion of the tissue is mainly populated by fibroblast-like cells surrounded by a dense connective tissue^8^. The third population, located in the superficial zone of the meniscus, possess a flattened, fusiform morphology. These cells are suggested to be potential progenitor cells with therapeutic and regenerative properties^9^. Previous studies have characterised these meniscal cell phenotypes and gene expression profiles in normal human and animal tissues^10–12^. However, to the best of our knowledge, the detailed phenotype of degenerate tissue-derived populations and the surface marker profile of the meniscal cells from the different regions of degenerate menisci have yet to be characterised.

Though human lateral and medial meniscus both are roughly semilunar and wedge-shaped, they have distinct dimensions and morphologies^7^. The lateral menisci display a greater variety in shape, size and thickness than the medial menisci^13^. It also covers a larger proportion of the tibial plateau (59 ± 6.8% laterally) in comparison to the medial meniscus (50 ± 5.5% medially)^14^. However, because of the lack of regular availability of the medial meniscus in degenerate knees, in this study we have only focused on investigating the lateral menisci from the medial compartment of OA patients who underwent total knee replacement (TKR) surgeries.

Cell-based meniscus tissue engineering is considered to represent a promising strategy for meniscus repair and regeneration^15^. Meniscal cells from normal human and animal menisci have demonstrated chondrogenic capacity in vivo and vitro^16,17^. However, human meniscal cells derived from the debrided tissue of bucket handle tears showed chondrogenic capacity in pellet culture with significantly less collagen type II and more collagen type I production compared with human bone marrow-derived mesenchymal stem cells (MSCs)^16^. This study also demonstrated the successful repair of rabbit meniscus punch defects with autologous meniscal cells delivered in hyaluronan-gelatin scaffolds after 12 weeks. In another defect model, sheep meniscus defects implanted with autologous meniscal cells pre-seeded into a collagen meniscus implant (CMI) demonstrated increased extracellular matrix production and enhanced vascularization after 3 months compared to non-seeded CMI scaffold controls and meniscus-resected controls^17^, suggesting that implanted cells contributed to the improved repair noted. However, whether or not meniscal cells derived from OA tissues can retain this therapeutic potential is unclear.

The present study compares the cell characteristics, chondrogenic capacities and phenotypic markers of meniscal cells derived from the inner and outer lateral meniscus (avascular and vascular zones) as well as donor-matched articular chondrocytes from the lateral femoral condyle taken from patients with medial compartment OA knee joints. The study aims to further our knowledge regarding the pathological status of these tissues and to assess the potential of cells derived from OA meniscal tissues for regenerative purposes.

## Results

### Histological scoring and analysis of meniscus sections

**Figure 1:**
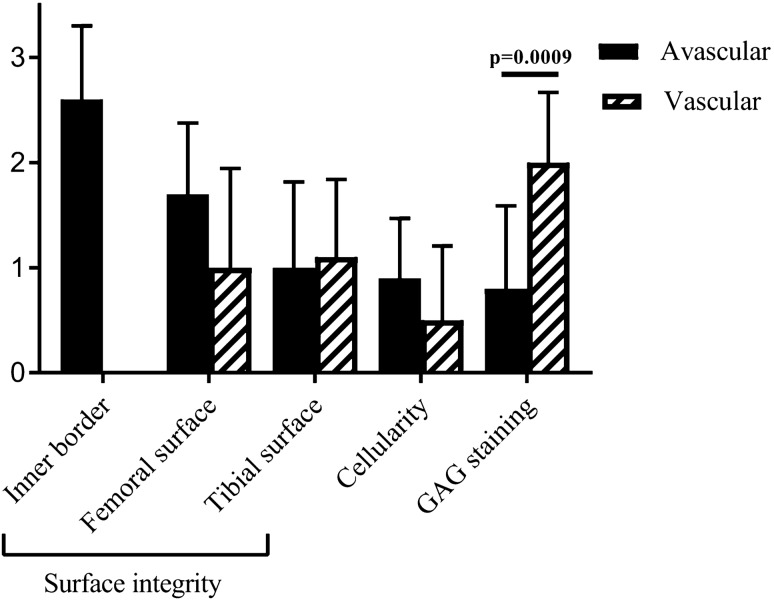
Histological grading results of avascular and vascular regions of the meniscus based on three scoring parameters (n = 10). The glycosaminoglycan (GAG) staining intensity in the vascular region was significantly higher than in the avascular region; (Grade 1: 0–3; Grade 2: 4–7; Grade 3: 8–11; Grade 4: 12–15). Data shown are the means ± the standard deviation.

Donors matched samples of cartilage, avascular and vascular meniscus tissue were obtained from 10 patients (6 males and 4 females, ages 46–87 years) undergoing TKR surgery (Table 1). Since the avascular and vascular zones often differed in their morphology and degree of degeneration, we decided to grade them separately. The metachromasia intensity in the matrix of the vascular zone was found to be significantly stronger than in the avascular zone of the tissue (Fig. 1). The inner borders scored either 2 or 3, compared to the other zones which generally had lower scores, suggesting that the inner rim of the meniscus in an OA joint was likely to be the first and most seriously affected structure in the disease process. There were no significant differences observed between regions for other histological parameters.

**Table 1:** Demographics of donors from which samples were sourced.

ID	Gender	Age	Meniscus	Microscopic grading
Donor 1	Male	46	Lateral	2
Donor 2	Female	53	Lateral	4
Donor 3	Female	66	Lateral	3
Donor 4	Female	66	Lateral	2
Donor 5	Male	66	Lateral	2
Donor 6	Female	67	Lateral	2
Donor 7	Male	69	Lateral	3
Donor 8	Male	69	Lateral	3
Donor 9	Female	75	Lateral	2
Donor 10	Female	87	Lateral	2

**Figure 2:**
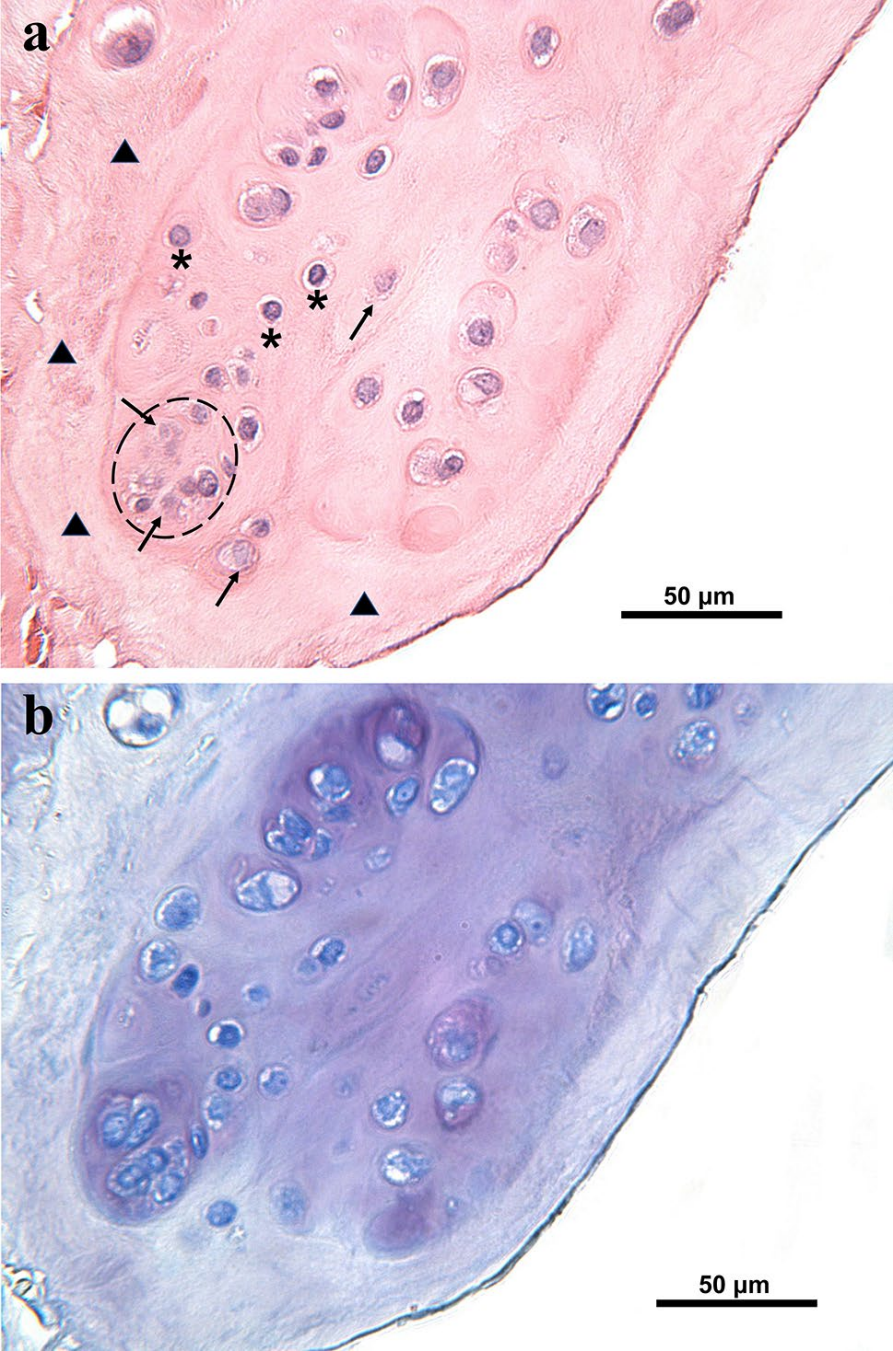
Representative meniscus histology from donor 9 stained with H&E and TB (avascular region, Grade 2): (**a**) Oedematous changes were observed in the meniscus surface zone, where the cells often appeared chondrocytic (*) sometimes forming clusters (dashed line). The area surrounding the oedematous region was typically acellular (▲) Note the necrotic appearance of some cells in this area and within the clusters (↗); (**b**) A higher intensity of TB staining was observed in the oedematous region.

Several noteworthy histological observations were made. Fibrillation, disrupted tissue structures and/or abnormal cellularity was noted mainly along the inner border, but less frequently elsewhere. These changes appeared to coincide with oedematous changes within the matrix close to surface (Fig. 2a). Cells in the swollen oedematous region showed a chondrocytic appearance. Cell clusters were also observed in these regions and the surrounding extracellular matrix was typically acellular. In addition, the toluidine blue (TB) intensity in the corresponding regions appeared stronger (Fig. 2b).

**Figure 3:**
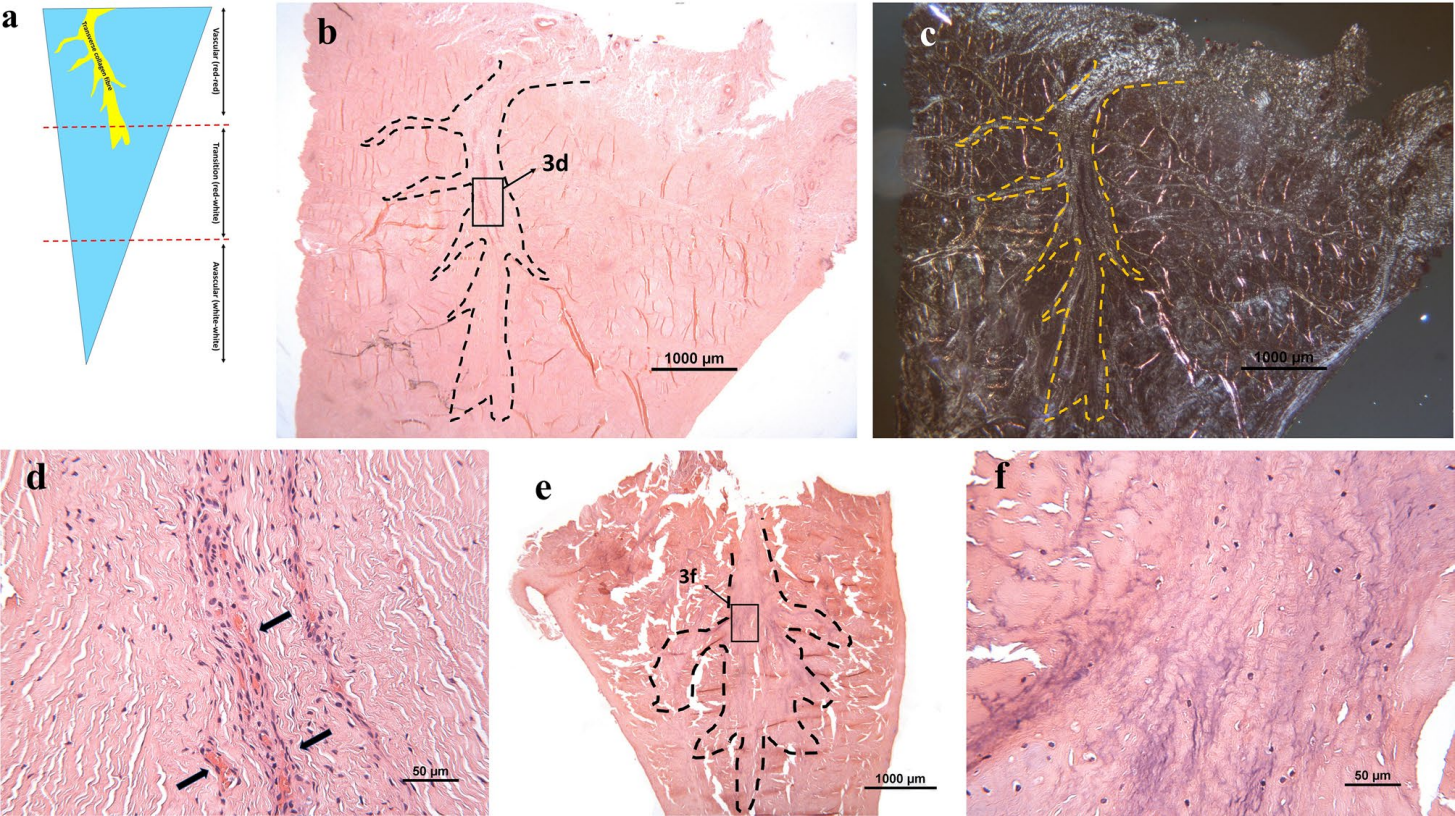
Representative images of Grade 2 (b-d: donor 4) and Grade 3 (e, f: donor 9) menisci with H&E staining: (**a**) The cross-section diagram of meniscus. The “tree-like” transverse collagen fibre (yellow) runs radially from synovial tissue into vascular region. (**b**) The collagenous ligament derived from the capsule presented a “tree-like” structure (dotted line); (**c**) The “tree-like” structure (dotted line) shown in (**b**) could be visualized easier under polarized light; (**d**) Blood vessels (arrows) were distributed along the “tree” root; (**e**) In the Grade 3 menisci where the matrix was fragmented; (**f**): The “tree-like” structure was more degenerate and without blood vessels in the Grade 3 menisci.

A collection of transverse collagen fibres, referred to as the transverse ligament, were found running from the synovial edge (Fig. 3a) into the vascular region. The ligament presented a “tree-like” formation of fibres interwoven into the avascular region in all 10 patient samples (Fig. 3b), which was most obvious when viewed under polarized light (Fig. 3c). Blood vessels in all six Grade 2 menisci were found associated with this ligament, running alongside the collagen fibres (Fig. 3d). Interestingly, in the Grade 3 and 4 menisci, less blood vessels were found associated with this “tree-like” structure compared to Grade 2 menisci (Fig. 3e,f).

### Growth kinetics

**Figure 4:**
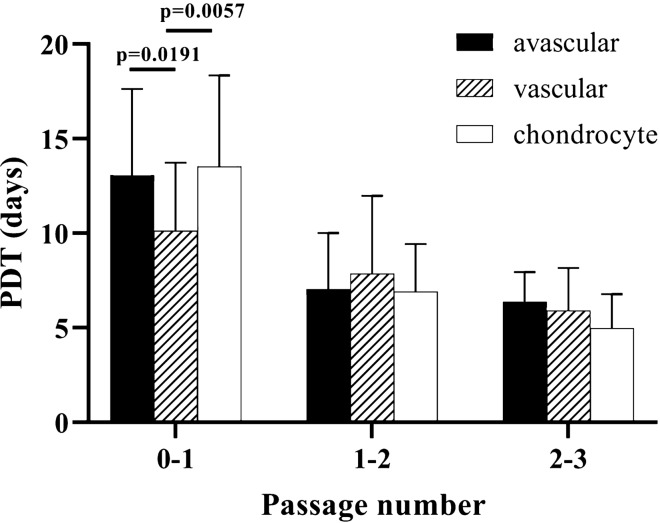
Graph to show the population doubling time (PDT) of avascular, vascular meniscal cells and chondrocytes during cell culture relative to passage number (passage 0–3). Data shown are the means ± the standard deviation**.**

Vascular meniscal cells proliferated at a significantly higher rate at passage 0–1 compared to avascular meniscal cells (p = 0.0191) and chondrocytes (p = 0.0057) (Fig. 4). Cell population doubling time (PDT) appeared to decrease with increased passage number. However, no significant differences were observed between passage 1–2 and passage 2–3.

### Cell surface marker expression levels at passage 0 and prior to chondrogenic differentiation at passage 2: donor-matched analyses of avascular and vascular meniscal cells and chondrocytes

**Figure 5:**
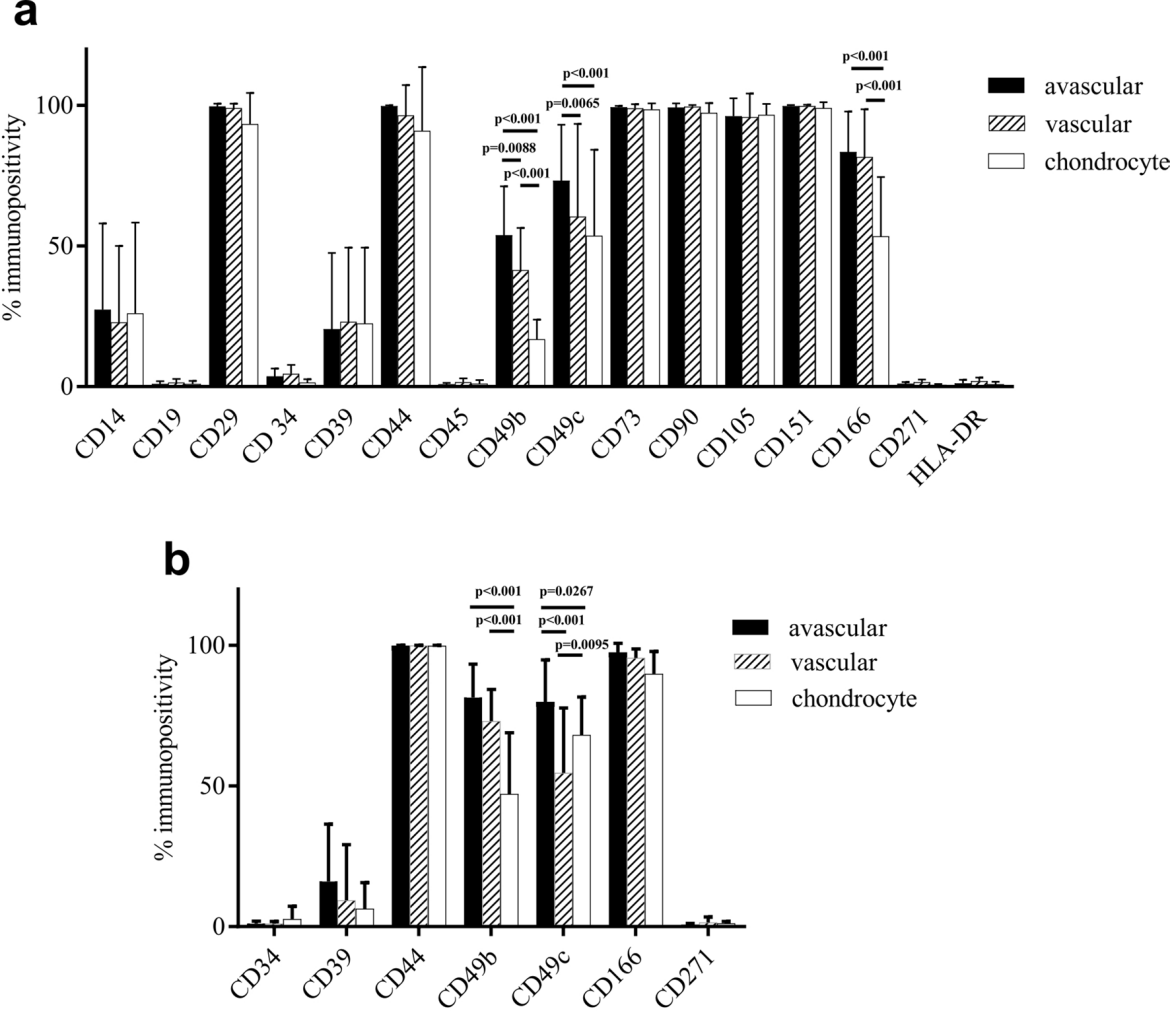
(**a**) Meniscal cells and chondrocytes exhibited similar immunopositivity for most of the markers investigated at passage 0. A greater percentage of meniscal cells were positive for CD49b, CD49c and CD166 compared with chondrocytes (10 donors); (**b**) A similar pattern was found for CD49b and CD49c immunopositivity across the cell types at passage 2 (6 donors). Data shown are the means ± the standard deviation.

For all three cell types and for all 10 donors at passage 0, the immunopositivity of the MSC markers CD73, CD90, CD105 was over 95%, which adhered to the International Society for Cellular Therapy (ISCT) criteria; however, around 25% of these cells were CD14 positive which should be lower than 2% in MSCs (according to the ISCT)^18^, but is similar to levels we have reported previously in MSCs derived from other musculoskeletal tissues. CD29 (integrin β1) and CD44 (hyaluronate receptor) were highly immunopositive on all cell fractions, being over 95% positive in avascular and vascular meniscal cells and over 90% positive on chondrocytes. CD19 (B lymphocyte antigen), CD34 (haematopoietic progenitor cell antigen), CD45 (protein tyrosine phosphatase receptor type C), CD271 (low-affinity nerve growth factor receptor) and HLA-DR (human leukocyte antigen-DR) were consistently below 5%, with no significant difference between groups. However, there was a significant difference in CD49b (integrin α2), CD49c (integrin α3) and CD166 (activated leukocyte cell adhesion molecule) immunopositivity between the three cell types (two-way ANOVA) (Fig. 5a). Similar patterns were found in the 6 donors tested at passage 2, prior to chondrogenic differentiation, with significant differences observed for CD49b and CD49c, but not CD166 (Fig. 5b). For CD49b, at passage 0, the immunopositivity was highest for the avascular cells (53.89 ± 17.41%) and lowest for the chondrocytes (16.80 ± 7.03%) with 41.46 ± 14.95% of cells from the vascular region being immunopositive. In passage 2, levels of CD49b on the three cell fractions followed a similar trend (avascular: 81.47 ± 11.88%, vascular: 73.03 ± 11.36%, chondrocyte: 47.16 ± 21.81%), but the difference between avascular and vascular cells was not significant. CD49c was 73.30 ± 19.84% immunopositive on avascular meniscal cells at passage 0, which was significantly higher than vascular meniscal cells (60.47 ± 32.99%) and chondrocytes (53.69 ± 30.60%). At passage 2, the immunopositivity of avascular meniscal cells for CD49c was again significantly higher than for the other cell populations, but those from the vascular region were significantly lower than chondrocytes (avascular: 79.99 ± 14.91%, vascular: 54.70 ± 23.04%, chondrocyte: 68.19 ± 13.46%). CD166 positivity was significantly higher on avascular (83.47 ± 14.41%) and vascular (81.68 ± 16.95%) meniscal cells compared to chondrocytes (53.47 ± 21.09%) at passage 0. However, time in culture appeared to upregulate CD166 on chondrocytes, from a mean of 53.47% at P0 to 89.82% at P2. No significant differences were observed for CD166 at passage 2 across the different cell populations (avascular: 97.50 ± 3.26%, vascular: 95.50 ± 3.22%, chondrocyte: 89.82 ± 7.97%).

### Comparing flow profiles and histological analyses

The immunopositivity of six markers was significantly related to some histological parameters scored in the avascular and vascular regions (Table 2). Analysis via the Jonckheere-Terpstra test revealed that when the meniscus tibial surface was more severely disrupted in the avascular region, the median number of avascular meniscal cells which were immunopositive for CD49b (*p* = 0.009) and HLA-DR (*p* = 0.028) increased. In avascular meniscal cells CD49b positivity was also found to be increased when the inner border had more severe disruption (*p* = 0.018); the same relationship was found with CD29 (*p* = 0.047). Further, when more hypocellularity was observed in the vascular zone, the median immunopositivity for CD34 and CD39 in vascular meniscal cells increased (*p* = 0.005 and *p* = 0.049, resp.). Finally, it was shown that as GAG intensity increased in the vascular zone, more of the vascular meniscal cells were immunopositive for CD19 (*p* = 0.024).

**Table 2:** Correlation between surface markers and histology scores.

	Region	Tibial			Inner border			Cellularity			GAG intensity		
		*p*	z	r	*p*	z	r	*p*	z	r	*p*	z	r
CD19	Avas	0.292	1.054	0.333	0.313	− 1.008	− 0.319	0.093	1.681	0.532	0.846	− 0.194	− 0.061
	Vas	0.492	0.688	0.218	–	–	–	0.756	0.311	0.098	***0.024***	***2.261***	***0.715***
CD29	Avas	0.288	1.064	0.336	***0.047***	***1.986***	***0.628***	0.619	− 0.497	− 0.157	0.667	− 0.430	− 0.136
	Vas	0.598	− 0.527	− 0.167	–	–	–	0.657	− 0.445	− 0.141	0.321	− 0.992	− 0.314
CD34	Avas	0.151	1.437	0.454	0.093	1.681	0.532	0.911	0.112	0.035	0.699	− 0.387	− 0.122
	Vas	0.280	1.080	0.342	–	–	–	***0.005***	***2.800***	***0.885***	0.100	1.644	0.520
CD39	Avas	0.151	1.437	0.454	0.313	1.008	0.319	0.575	− 0.560	− 0.177	0.699	− 0.387	− 0.122
	Vas	0.202	1.277	0.404	–	–	–	***0.049***	***1.970***	***0.623***	0.681	0.411	0.130
CD49b	Avas	***0.009***	***2.595***	***0.821***	***0.018***	***2.360***	***0.746***	0.911	0.112	0.035	0.627	− 0.486	− 0.154
	Vas	0.280	− 1.080	− 0.342	−	−	−	0.756	0.311	0.098	0.537	− 0.617	− 0.195
HLADR	Avas	***0.028***	***2.203***	***0.697***	0.911	0.112	0.035	0.575	0.560	0.177	0.561	− 0.581	− 0.184
	Vas	0.377	0.884	0.280	–	–	–	0.254	1.141	0.361	0.150	1.439	0.455

Jonckheere-Terpstra test: Avascular region (marked with grey background); vascular region (marked with white background). Only values that have significant differences are shown in the Table; see the full dataset in the Supplementary Table 1. The significant values are highlighted in bold and italics.

### Gene expression profiles: donor-matched analyses of avascular and vascular meniscal cells and chondrocytes

**Figure 6:**
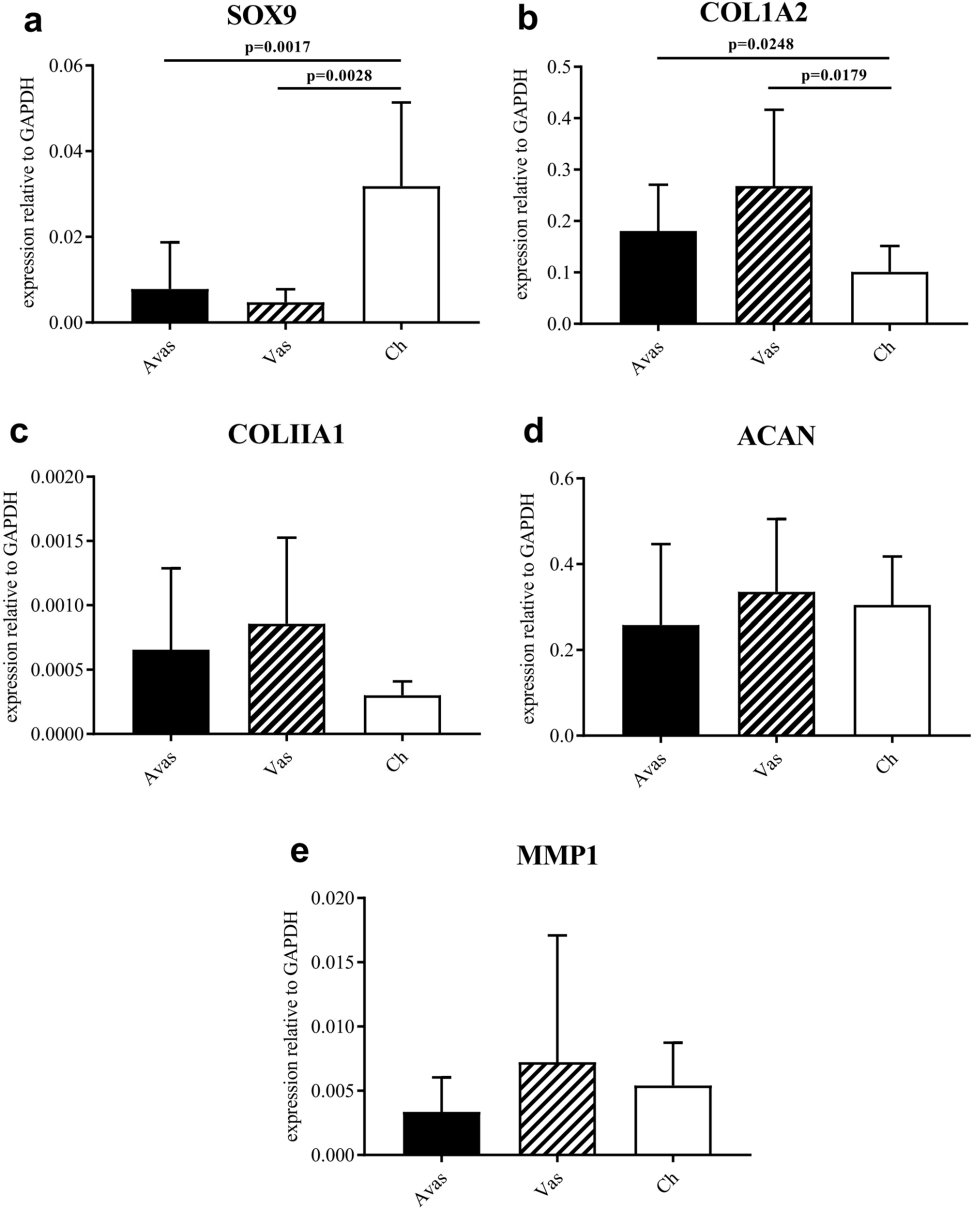
The chondrogenic genes and MMP-1 expression profiles in Avas, Vas and Chondrocytes after 14 days in monolayer culture (**a**–**e**). Data shown are the means ± the standard deviation of triplicate technical replicates and 10 donors for each cell population. Gene expression is shown relative to the reference genes, glyceraldehyde-3-phosphate dehydrogenase (GAPDH).

A significantly higher expression level of SOX-9 was found in chondrocytes compared to avascular and vascular meniscal cells (Fig. 6a). Unsurprisingly, the avascular and vascular groups showed a higher expression level of collagen type I compared to donor matched chondrocytes (Fig. 6b). No significant differences were found in the expression levels of collagen type II (COL II), aggrecan (ACAN) or MMP-1 between the cell types (Fig. 6c-e).

### In vitro chondrogenic pellet analysis

**Figure 7:**
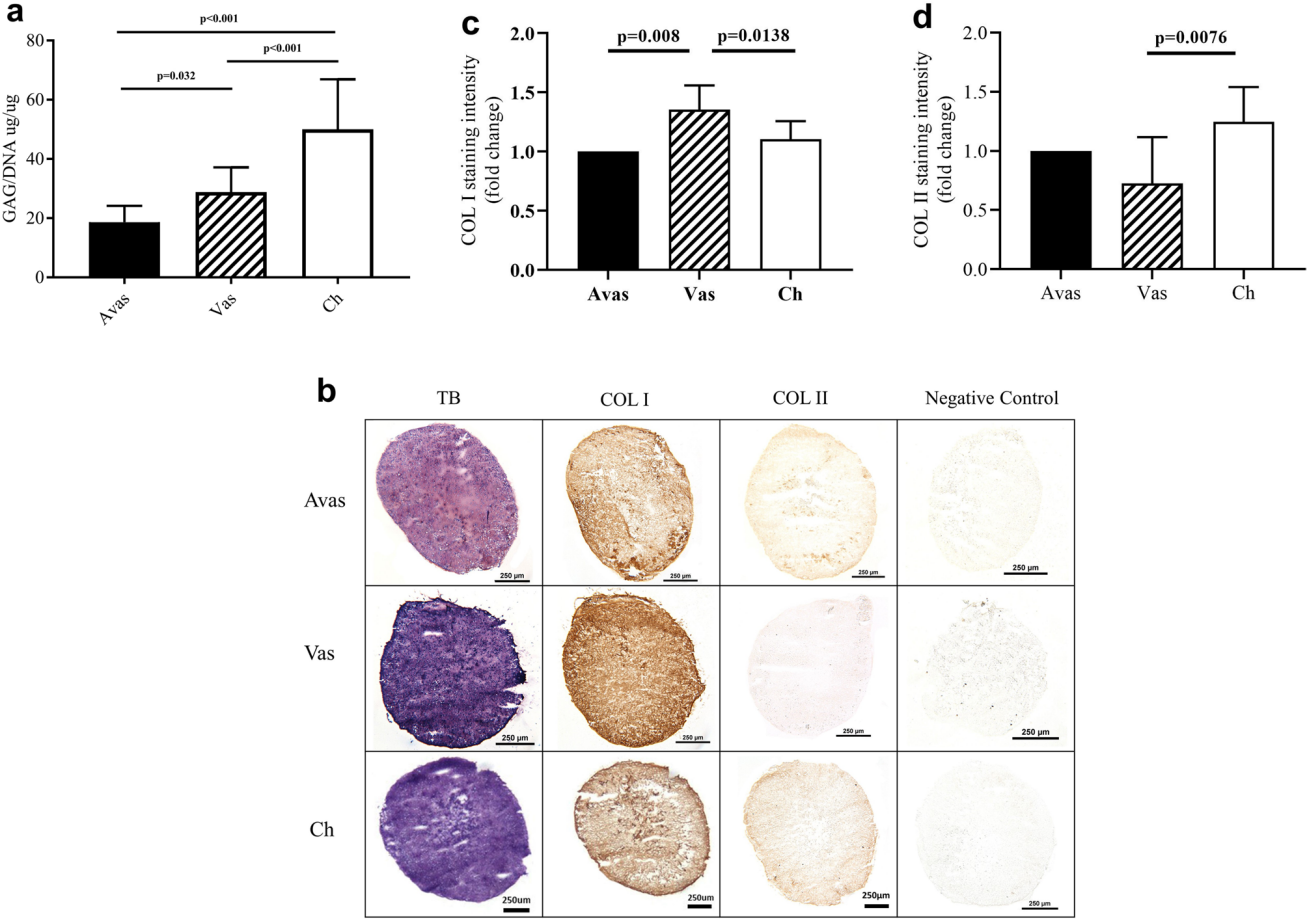
The chondrogenic assessment of avascular and vascular meniscal cells and chondrocytes. (**a**) GAG/DNA quantitation after 28 days of culture in pellets, a comparison of Avascular (Avas) and Vascular (Vas) meniscal cells and chondrocytes (Ch). Data shown are the means ± the standard deviation of triplicate runs and 6 donors for each cell population. (**b**) Histological analysis of a representative donor 6, chondrogenic pellet sections from Avas, Vas, Ch showing toluidine blue (TB), collagen type I (COL I) and collagen type II (COL II) staining. Scale bars represent 250 μm. (**c**) Collagen type I and (d) collagen type II semi-quantitative immunohistochemical (IHC) analysis, relative fold change to avascular meniscal pellet.

Chondrogenic capacity across all of the cell types was tested in 6 donors after 28 days of chondrogenic differentiation in pellet culture. In terms of GAG/DNA analyses, chondrocytes consistently produced the highest levels of GAG, while avascular meniscal cells showed lower GAG levels compared to vascular meniscal cells (Fig. 7a). This finding appeared to match the histological grading of TB intensity in the avascular and vascular zones: that is, the vascular regions demonstrated more pronounced matrix metachromasia compared to avascular regions (Figs. 1a, 7a). The donor-matched chondrogenic pellets showed variable chondrogenic capacity across individuals. Overall, strong collagen type I staining was observed in all pellets and across cell types after 28 days of chondrogenic induction, with the highest staining intensity in the vascular meniscal cells (Fig. 7b,c). This observation appeared to match the collagen type I gene expression profile for each cell population (Fig. 6b). However, weak collagen type II staining were detected in all cell types, with a significantly stronger staining intensity produced by chondrocytes pellets compares with vascular meniscal cell pellets (Fig. 7d).

### Chondrogenic potency analysis

Multilevel modelling analysis was conducted to identify chondrogenic potency predictors prior to chondrogenic differentiation (Table 3). CD49b immunopositivity positively associated with GAG quantitation (*p* = 0.035). Similar to previous chondrogenic analysis (Fig. 7a), cell types (vascular meniscal cells and chondrocytes) had a significant impact on GAG quantitation compare to avascular meniscal cells (*p* = 0.003 and *p* < 0.001, resp.). This analysis also showed that CD44 expression negatively associated with GAG quantitation in pellet cultures (*p* = 0.011).

**Table 3:** Multilevel modelling.

	Coefficient	95% confidence interval		*p*
		Lower	Upper	
CD34	− 0.4543	− 1.8915	0.983	0.553
CD39	0.277	− 0.0109	0.565	0.096
CD44	− ***77.5159***	− ***123.7309***	− ***31.301***	***0.011***
CD49b	***0.4098***	***0.0922***	***0.727***	***0.035***
CD49c	0.0606	− 0.1577	0.279	0.601
CD166	− 0.2386	− 0.6014	0.124	0.233
CD271	− 2.911	− 6.1629	0.341	0.117
Vascular-avascular	***21.0973***	***11.0721***	***31.122***	***0.003***
Chondrocytes-avascular	***44.3104***	***29.7228***	***58.898***	** < .001**

The significant values are highlighted in bold and italics.

## Discussion

It is widely accepted that meniscus degeneration leads to the deterioration of articular cartilage and the onset of osteoarthritis^7^. However, the precise mechanisms of the early meniscus degeneration process are still unclear, particularly in terms of cellular phenotypic changes and extracellular matrix alterations^19^. We designed this study to undertake a comprehensive characterisation of meniscal cell populations derived from the lateral menisci obtained from TKR patients. Although we were careful to choose regions with an intact morphology, these tissues were no doubt affected by an OA pathological environment for a prolonged period. In the medial compartment of OA patients, the increased medial loading disrupts the normal medio-lateral load sharing balance. This imbalance leads to lateral compartment lift-off^20^. Therefore, the lateral meniscus experiences abnormally lower loading which slows down its tissue breakdown^21^. Based on our histological findings, the lateral menisci derived from such joints have experienced varying degrees of deterioration as was demonstrated by our use of the Pauli et al. histological grading system^4^.

Petersen et al. first described the loosely arranged collagen fibres entering the external circumference of the meniscus, originating from the joint capsule and inserting between the circular fibre bundles of the meniscus^22^. In our study, these “tree-like” shaped structures were identified in all donors. However, to our knowledge, no previous study has described them in relation to the pathological changes observed over time with knee OA. Arnoczky et al. (1982) demonstrated small radial branch of blood vessels which came from the perimeniscal capillary plexus penetrating the meniscal stroma a short distance into the main body of the meniscus^23^. The formation of these vessels was seen to associate with the “tree-like” collagen fibres entering into the meniscus. In the present study, histological analyses showed that these blood vessels mainly appear along the collagen fibre “tree root”. However, the vessels were only found in the “tree root” in the vascular area of Grade 2 menisci and were rarely present in the same region in Grade 3 or 4 specimens. This result matches findings from a previous study, which reported blood vessel occurrence only in the dense connective tissue but not in the fibrocartilage^24^. In this study it was also reported that only a quarter of the meniscus tissue was vascularised in the aged meniscus, whereas the outer one third of the meniscus tissue was vascularised in young adult menisci. Degenerative meniscal tears generally have a complex pattern and are mainly found in the middle and posterior body of the meniscus^19^. Partial meniscectomy or non-operative management is normally chosen to treat these patients due to the low healing potential of the degenerate meniscus^25^. The diminished blood supply noted in the vascular region of the degenerate meniscus may play an important role in its reduced self-healing capacity.

The meniscal cells from the OA joint consist of a heterogeneous population, which has not been well characterised previously. In our study, similarities found in the expression of surface molecules (CD14, CD19, CD29, CD34, CD39, CD44, CD45, CD73, CD90, CD105, CD151, CD271, HLA-DR) from avascular and vascular meniscal cells and chondrocytes indicate their overlapping characteristics and chondrogenic potential. Despite these similarities, differences in CD49b (integrin α2), CD49c (integrin α3) and CD166 (ALCAM) were noted between all cell fractions derived from OA affected tissues. Grogan et al. (2017) reported, in the normal human meniscus, a greater percentage of meniscal cells that were positive for CD14 (LPS-receptor), CD26 (dipeptidyl peptidase IV) and CD49c compared to articular chondrocytes (10 donors)^10^. The differences noted in our study in comparison indicate that the OA environment influences the cell surface molecule expression in meniscus and cartilage.

Grogan et al. highlighted that there was positive immunostaining for CD166 (activated leukocyte cell adhesion molecule, ALCAM) on cells that predominately surrounded the blood vessels in the vascular region of the meniscus and on cells at the meniscus surface^10^. These cells could be progenitors, as CD166 has previously been used to identify the progenitor populations from chondrocytes in healthy cartilage^26^. A previous study demonstrated a higher number of CD34 (a stem cell marker) and CD146 (a pericyte marker) positive freshly isolated cells to be found in the vascular region compared with the avascular region of the lateral meniscus in OA patients. This CD34 and CD146 positive cell populations showed multilineage differentiation capacities and contributed to meniscus repair in a rat model^27^. Our flow cytometry analysis of the different meniscal regions showed higher expression levels of CD166 in the vascular meniscal cell fraction. In addition, our PDT data demonstrated that the vascular meniscal cells possess a higher proliferation rate compared to the other cell types. Together this indicates the presence of progenitors associated with the blood vessels or perhaps that the vasculature in the peripheral region drives a more progenitor-like meniscal cell phenotype in this region. This theory is further supported by our hypothesis that the “tree-like” fibres which “tie” the synovial tissue and meniscus together, not only provide the structural supports for the blood supply into the peripheral meniscus, but may also harbour a conduit for the “progenitor” cells which could originate from synovium, as CD166 positive mesenchymal progenitor populations have been identified in the synovium tissue of osteoarthritic knees^28^. Ideally, the use of fluorescence-activated cell sorting (FACS) to isolate and quantify CD166 immunopositive populations for further progenitor phenotypic characterisation would be required to validate our hypothesis in future work. In the more degenerate meniscus, we noted the absence of blood vessels with “progenitor” cells in these “tree-like” fibres. We hypothesise that this structure (comprising of collagen fibres, blood vessels and cells) plays an important role in maintaining the meniscus matrix, protecting against the degeneration process in the early OA stages; however, this requires further investigation before firm conclusions may be drawn. In addition, the “tree-root” cell population may have regenerative properties pertinent to the development of meniscus tissue engineering strategies.

Integrins play a key role in mediating chondrocyte-ECM interactions in the OA pathophysiology of articular cartilage degeneration^29^. However, the role that integrins have in the degeneration of the meniscus is still unclear. CD49b and CD49c are integrin alpha subunits which were first identified as ECM receptors for collagens, laminins and fibronectin^30^. CD49b was found to have an increased expression in cartilage in the late stage OA mouse model^31^. Unusually, in this animal study CD49b cell signalling was found to be induced by changes in the ECM components which increase the catabolic activity of chondrocytes and favour cell death, as a consequence of increased metalloproteinase (MMP) activity. Integrin α3 was also identified to be differentially expressed in the whole knee joint in the destabilization of the medial meniscus (DMM) mouse model, which was closely associated with the development of OA^32^. Our flow cytometry results demonstrate that CD49b and CD49c positivity levels are higher in avascular meniscal cells compared with vascular meniscal cells and chondrocytes in both passage 0 and passage 2 cell populations. This result might be due to the fact that fraying of the avascular region was found in all of the samples included in the study, perhaps indicating a more advanced response to OA progression in the avascular region of the meniscus compared to the other regions examined. The correlation results also demonstrated that with more disrupted tissue structures in the meniscus inner border, the avascular meniscal cells’ immunopositivity for CD49b and CD29 (integrin β1) increased. β1 integrin-collagen interaction is a critical signalling pathway for chondrocyte survival, which prevents apoptosis^33^. Therefore, our results could support the hypothesis that the OA-like ECM changes in the avascular region of the meniscus induced the up-regulation of CD49b, CD49c and CD29, although additional mechanistic study will be required to draw firm conclusions.

The multilevel modelling analysis performed to find predictors of chondrogenic potency in this study suggested that higher expression levels of CD49b were significantly associated with higher GAG quantities. A previous study demonstrated that gene expression levels of CD49b were up-regulated in human chondrocyte pellet cultures at day 14 compared to monolayer cultures^34^. Another study has shown that GAG production significantly increases over time in pellet cultures compared to monolayer cultures^35^. However, CD49b expression pre-pellet formation has not previously been shown to associate with higher post-pellet GAG levels. Although the higher expression of CD44 was also found to correlate with GAG production, when interpreting this finding it should be kept in mind that in our experiments the CD44 expression ranged from 99.6% to 100% (Fig. 5b). This represented a variation of 0.4%, similar in magnitude to the accuracy by which CD44 could be determined by flow cytometry (Supplementary Table 2). As a consequence, this significant association could well be a “false positive”, finding an effect when none actually exists. Multiple regression models can be prone to this type of error^36^. In addition, such a small variation in CD marker expression is unlikely to have any biological effect. Therefore, CD44 was not considered as a marker associated with GAG production in this case. We should also be cautious when interpreting any results derived from a small sample size. We acknowledge this as a limitation of the study.

Tears of the meniscus are a common sporting injury in fairly young individuals. If they can be repaired biologically, this could avoid the development of OA which is otherwise likely to occur. Autologous meniscal cells derived from the meniscus lesion site could represent a potential cell source for meniscus repair strategies^7^. In the current study, we have shown that extracted and sub-cultured avascular and vascular meniscal cells possess similar chondrogenic capacity with a higher gene expression level of collagen type I compared to donor matched chondrocytes, which more closely matches each of the tissue’s native collagen composition. As such meniscal cells may represent a more desirable cell source for cell therapy of the meniscus as they retain their capacity to more specifically reconstitute the native meniscus tissue matrix composition. Such an approach would require two surgical interventions including a biopsy to extract meniscal cells and a second procedure to implant cells or a tissue-engineered meniscus. Moreover, insufficient cell numbers may be obtained from limited “normal” donor tissue. To address these potential issues, human bone marrow stromal cells (BMSCs) were investigated as a supplemental or alternative cell type to meniscal cells for meniscus tissue engineering. Studies have shown that co-culturing BMSCs with meniscal cells resulted in enhanced chondrogenic ECM production under normal and low oxygen in vitro conditions^37^. Hagmeijer et al. (2018) also demonstrated the feasibility of a one stage procedure by using a rapid digestion for autologous meniscal cells combined with allogenic BMSCs (20:80), applied to a commercial Collagen Meniscus Implant (CMI) scaffold with fibrin glue as carrier in a cadaveric study^38^. Current treatments for meniscus deficient patients with knee pain that has developed several years after meniscectomy are partial meniscus replacement with a biodegradable scaffold or meniscus allograft transplantation, both of which produce sub-optimal clinical outcomes^39,40^. The ideal solution is to perform a single stage cell-based partial meniscus replacement when meniscectomy is decided to be the surgical course of action. In this case the surgeon would be able to obtain sufficient meniscus tissue for autologous meniscal cell derivation. Moreover, it is expected that the patient will observe improved outcomes when they receive the treatment at an early stage of disease rather than as a remedial course of action years after the development of OA.

There are several limitations associated with the current study that should be acknowledged. Firstly, we have focused solely on the lateral meniscus from the medial compartment in OA patients, a comparison of medial menisci from lateral OA patients would make for a more complete study. However, insufficient samples for this comparison could be obtained because knee OA is more commonly seen in medial rather than lateral compartment^41^. Another limitation of this study is that RNA extraction was not performed to check the gene expression levels of post-chondrogenic pellet but only in monolayer culture because of limited cell numbers. Such chondrogenic related gene expression analysis would complete the chondrogenic potency analysis undertaken in the study.

In conclusion, our study has indicated that CD49b, CD49c and CD166 appear to be important phenotypical markers which can discriminate cells from avascular or vascular meniscus and cartilage in the OA joint. We have observed distinct meniscal cell profiles, which are reflected in corresponding changes in the tissue’s structure histologically. The meniscus “tree-like” structure of collagen fibres observed throughout the histological analyses in this study may play an important role in supporting the blood supply to vascular region of meniscus and maintaining the meniscus integrity, protecting against the structural breakdown which can occur as OA progresses. We have also demonstrated that meniscal cells derived from the lateral meniscus of medial compartment OA patients have chondrogenic capacity in vitro and hence could represent a potential cell source to consider for meniscus tissue engineering.

## Methods and materials

### Lateral meniscus and cartilage harvest

All the tissue samples used in this study were obtained following the provision of written, informed consent from patients. Favourable ethical approval was given by the National Research Ethics Service Committee Northwest Liverpool East (11/NW/0875) and all experiments were performed in accordance with relevant guidelines and regulations. Macroscopically, intact lateral menisci from the lateral compartment, as well as donor-matched articular cartilage from the lateral femoral condyle, which demonstrated minimal OA changes (macroscopically classified as Outerbridge Grade I or II), were harvested from donors who were undergoing TKR for medial compartment (n = 10; mean age 66.4 ± 11.1; age range 46–87 years; 4 males, 6 females).

### Cell isolation

**Figure 8:**
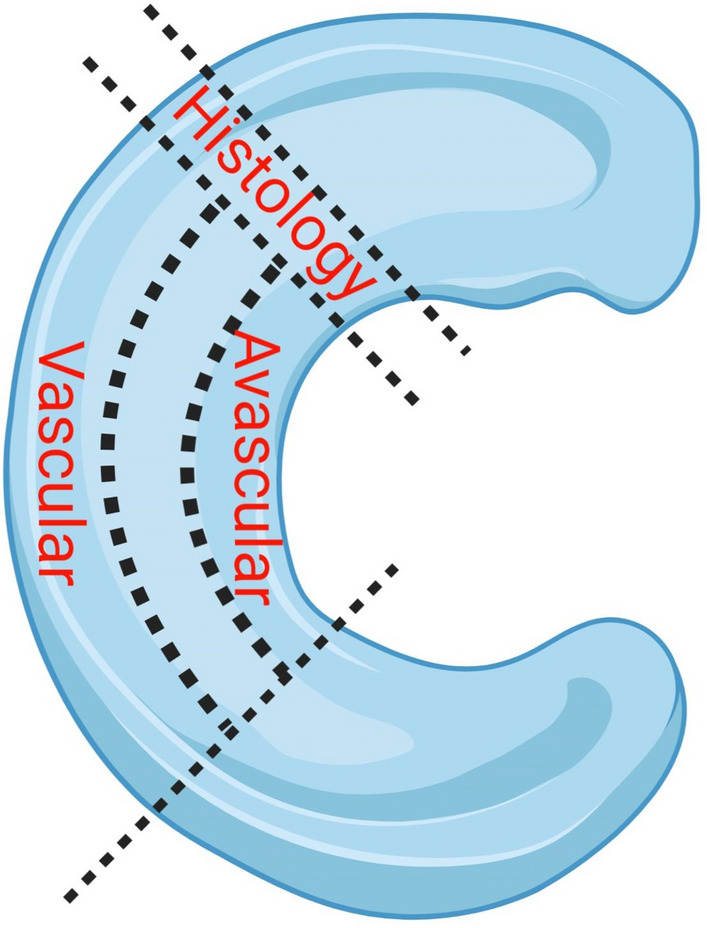
Schematic diagram of the process used for meniscus dissection: The medial body of the macroscopically “normal” meniscus was longitudinally divided into three parts: the inner third was used to extract avascular meniscal cells, the outer third for the extraction of vascular meniscal cells. The adjacent section which composed of the inner and outer regions was dissected for histological grading. Figure was created with BioRender.com.

The middle one third of the lateral meniscus was dissected equally into the inner (avascular zone), middle and outer (vascular zone) in a longitudinal direction (Fig. 8). The middle portion was discarded such that only the definite vascular and avascular zones were studied as such. A section of the meniscus adjacent to that used for cell culture experiments was obtained for histology. Meniscus and cartilage tissues were digested in type II collagenase (245U/ml; Worthington, USA) in Dulbecco’s Modified Eagle Medium (DMEM)/ F12 (1:1) (Gibco, USA) and 1% penicillin–streptomycin (P/S) (ThermoFisher Scientific, USA) for 16 h at 37 °C. The digested tissues were filtered through 70 μm cell strainers (ThermoFisher Scientific, USA) and cells were seeded at a density of 5000 cells per cm^2^. The meniscal cells were maintained in monolayer culture in a humidified environment of 5% (v/v) CO_2_ and 37 °C for 14 days.

### Growth kinetics

Population doubling times (PDT) were calculated for each cell type (from passage 0–3) using the following formula: PDT = (t_2_ − t_1_) × ln(2)/ln(n2/n1), t_1_ = the time of cell seeding, t_2_ = the time of cell harvest and n = the cell population at the matching time points.

### Histological analysis of meniscus sections

The region of the meniscus adjacent to that used for cell culture from 10 donors was fixed in 10% neutral buffered formalin and embedded in paraffin. Sections  (4 μm thick) were stained with haematoxylin and eosin (H&E) for the visualisation of morphological details and with toluidine blue (TB; British Drug Houses, UK) to assess the GAG distribution. All samples were categorised using a modified microscopic meniscus grading system developed by Pauli et al.^4^. Briefly, this modified grading system scored menisci based on the following parameters: (A) Meniscus surface integration of the femoral and tibial side and at the inner border (0: smooth, 1: slight fibrillation and undulation, 2: moderate fibrillation, clefts and undulation, 3: severe fibrillation, clefts and undulation); (B) Cellularity (0: normal, 1: diffuse hypercellularity, 2: diffuse hypo/acellular regions, 3: hypocellularity, empty lacuna, pyknotic cells); (C) Toluidine blue matrix staining intensity (0: none, 1: slight, 2: moderate, 3: strong). The avascular and vascular regions were scored separately by two independent (blinded) assessors. The total score was calculated as follows: S_total_ = ((A + B + C)_Avas_ + (A + B + C)_Vas_)/2 + A_inner-border_. The mean score of the two individual readers was converted to the following grades: Grade 1 represents normal tissue (score 0–3) and Grade 2 indicates mild degeneration (score 4–7). Moderate degeneration is seen in Grade 3 tissue (score 8–11), while Grade 4 represents the most severe degeneration (score 12–15).

### Flow cytometry

After 14 days of monolayer culture expansion (passage 0) each cell type was re-suspended in a PBS buffer of 2% (v/v) bovine serum albumin (BSA; Sigma-Aldrich). Flow cytometry receptors were blocked using a PBS buffer of 10% (v/v) human immunoglobulin (Grifols, Spain) at 4 °C for 1 h. Immunopositivity for 16 molecules which are indicative of mesenchymal stromal cell (MSC) profile (CD14, CD19, CD34, CD45, CD73, CD90, CD105, HLA-DR), chondrogenic potency or cell adhesion molecules (CD29, CD39, CD44, CD49b, CD49c, CD151, CD166, CD271) were targeted. At passage 2, prior to chondrogenic differentiation, a smaller flow panel including chondrogenic potency molecules (CD39, CD44, CD271) and those in which a marked difference was observed between cell populations at passage 0 (CD49b, CD49c, CD166) were investigated in 6 donors.

### RNA extraction and quantitative real-time polymerase chain reaction (qRT-PCR)

After trypsinisation at passage 0, 100,000 cells were centrifuged (350×*g* for 8 min), frozen in liquid N_2_, and stored at − 80 °C temporarily before mRNA extraction. mRNA was extracted using a RNeasy Mini Kit (Qiagen, Hilden, Germany) according to the manufacturer’s instructions. A High-Capacity cDNA Reverse Transcriptase Kit (Applied Biosystems, Warrington UK) was used for reverse transcription. qRT-PCR was performed on the Quant Studio 3 Real-Time Quantitative PCR System (Applied Biosystems) using a SYBR Green Reaction Mix. Gene expression levels of collagen type I (COL1A2), collagen type II (COLIIA1), aggrecan (ACAN), SOX-9 and matrix metalloproteinase-1 (MMP-1) were normalised against the housekeeping genes glyceraldehyde-3-phosphate dehydrogenase (GAPDH) (Qiagen, QuantiTect Primer Assay). The relative gene expression level of each gene was determined by using the comparative C_T_ method^42^.

### Chondrogenic differentiation

The chondrogenic potency of the three donor-matched cell populations were assessed at passage 2 using a well-established 3D pellet culture system in 6 donors^43^. Briefly, 2 × 10^5^ cells were centrifuged into a cell pellet with DMEM F12, P/S (1%), ITS (1%), ascorbic acid (0.1 mM) (Sigma-Aldrich), dexamethasone (10 nM), sodium pyruvate (Sigma-Aldrich) and transforming growth factor β-1 (TFG-β-1, PeproTech, London, UK) (10 ng/ml). After 28 days in culture, n = 3 pellets were used for biochemical GAG/DNA quantitation, n = 3 pellets were snap frozen in liquid nitrogen-cooled hexane and stored at − 80 °C prior to histological analysis.

### GAG/DNA analysis

Pellets were digested in papain to release GAG and DNA. The papain digestion buffer was composed of 50 mM sodium phosphate (BDH), 20 mM EDTA (Sigma-Aldrich), 20 mM N-acetyl cysteine (BDH) and adjusted to pH 6.0. Papain was added to the buffer to reach the final concentration of 125 µg/ml. Each pellet was digested in 200 µl of the papain solution at 60 °C for 3 h. Samples were centrifuged at 1000 g for 5 min and stored at -20 °C prior to use. The GAG content in pellets was measured by 1,9-dimethylmethylene blue (DMMB) assay^44^, with chondroitin sulphate (C9819, Sigma-Aldrich) from bovine trachea used to construct a standard curve. Briefly, 50 μl of each sample was added in duplicate wells of a 96 well plate, with 200 μl of DMMB dye. The results were read immediately at A_530nm_ and A_590nm_. The standard curve was plotted using the following equation: (A530nm/A590nm) − (A_530nm blank_/A_590nm bank_). The total GAG content of each pellet was calculated using the standard curve equation. The DNA content was measured spectrofluorometrically using the PicoGreen dsDNA Assay kit (Invitrogen) according to the manufacturer’s instructions. Finally, the amount of GAG measured in the chondrogenic pellet was normalised to its DNA content.

### Histological and immuno-histochemical analyses of pellets

Three pellets from each cell population were snap frozen in liquid nitrogen and stored at − 80 °C prior to use. Pellets were sectioned at a 7 μm thickness and collected onto poly-l-lysine–coated slides. Cryosections were stained with TB (BDH) to assess the general tissue morphology and GAG composition of the extracellular matrix. In addition, immunohistochemistry for collagens type I and II was undertaken. In brief, sections were incubated with ovine hyaluronidase (4800U/ml, Sigma, UK) prior to fixing in 10% formalin. Primary antibodies raised against collagens type I (1:500, clone I-8H5, MP Biomedicals, Cambridge, UK) or type II (1:50, clone CIIC1, DHSB, University of Iowa, USA) were incubated with sections for 1 h. After washing with PBS, sections were incubated with the secondary biotinylated antibody at 50 µg/ml (goat anti-mouse, VECTASTAIN ABC kit, Vector Laboratories, Peterborough, UK), which was added for 30 min. 0.3% hydrogen peroxide in methanol was used to block endogenous peroxidase activity. Enhanced labelling was performed with streptavidin-peroxidase (VECTASTAIN Elite ABC kit, Vector Laboratories, Peterborough, UK) and visualised with diaminobenzidine (DAB, ImmPACT, Vector Laboratories, Peterborough, UK), after the sections were dehydrated. The immunochemistry staining intensity of collagen type I and type II was quantified using ImageJ Fiji Software (version 1.2; WS Rasband, National Institute of Health, Bethesda, MD)^45^.

### Statistical analyses

All data were inputted into GraphPad Prism (Version 7.04, USA) and Jamovi (Version 1.1.9.0) for statistical analysis. Differences between cell types were assessed by performing one-way ANOVAs with Tukey’s multiple comparisons for population doubling time, positive percentage fluorescence signal, gene expression level, GAG/DNA comparisons and semi-quantitative of collagen type II IHC intensity. Two-way ANOVAs were used to compare the positive percentage fluorescence signal of different cell types and histological scores in avascular and vascular regions. The Jonckheere–Terpstra test was used to assess the correlation between the positivity of surface markers and meniscus histological scores. Multilevel modelling was performed to determine whether expressions of cell surface markers were associated with chondrogenic outcome as measured by GAG/DNA content. Cell source and cell surface marker positivity were considered as fixed effects, while the donor was considered as a random effect. Our lab previous data from flow marker reliability test (not published) was used to evaluate the reliability of chondrogenic predictors in multilevel modelling results (Supplementary Table 2). For all tests, values of *p* < 0.05 were considered statistically significant.

## Supplementary Information

Supplementary Information.
