# Supplementary material for: Characterization of regional meniscal cell and chondrocyte phenotypes and chondrogenic differentiation with histological analysis in osteoarthritic donor-matched tissues
Source: Sci Rep. 2020 Dec 10;10:21658. doi: 10.1038/s41598-020-78757-6 (PMC7730426; doi:10.1038/s41598-020-78757-6)
Supplement: Supplementary file 1 — Supplementary Information. [file 41598_2020_78757_MOESM1_ESM.pdf]

Supplementary Appendix

Characterization of Regional Meniscal Cell and Chondrocyte Phenotypes and Chondrogenic Differentiation with Histological Analysis in Osteoarthritic Donor-Matched Tissues

Jingsong Wang[1,2,3], Sally Roberts[1,2], Jan Herman Kuiper[1,2], Weiguo Zhang[4], John Garcia[1,2], Zhanfeng Cui[5], Karina Wright[1,2*]

1. Keele University, School of Pharmacy and Bioengineering, Keele, Staffordshire, ST5 5GB, UK
2. The Robert Jones and Agnes Hunt Orthopaedic Hospital NHS Foundation Trust, Oswestry, Shropshire, SY10 7AG, UK
3. Dalian Medical University, Dalian, 116044, China
4. Department of Orthopaedic Surgery, First Affiliated Hospital, Dalian Medical University, Dalian, 116011, China
5. Institute of Biomedical Engineering, Department of Engineering Science, University of Oxford, Oxford, OX1 3PJ, UK

Corresponding Author: Karina Wright (Email: karina.wright1@nhs.net)

Supplementary table 1: Correlation between Surface markers and Histology scores

	Region	Femoral			Tibial			Inner border			Cellularity			GAG intensity		
		p	z	r	*p*	z	r	*p*	z	r	*p*	z	r	*p*	z	r
CD14	Avas	0.764	0.301	0.095	0.924	0.096	0.030	0.093	1.681	0.532	0.575	0.560	0.177	0.846	0.194	0.061
	Vas	0.696	0.391	0.124	0.623	0.491	0.155	-	-	-	0.604	0.518	0.164	0.681	-0.411	-0.130
**CD19**	Avas	0.920	0.100	0.032	0.292	1.054	0.333	0.313	-1.008	-0.319	0.093	1.681	0.532	0.846	-0.194	-0.061
	**Vas**	0.328	0.977	0.309	0.492	0.688	0.218	-	-	-	0.756	0.311	0.098	***0.024***[*]	***2.261***	***0.715***
**CD29**	**Avas**	0.437	-0.777	-0.246	0.288	1.064	0.336	***0.047***[*]	***1.986***	***0.628***	0.619	-0.497	-0.157	0.667	-0.430	-0.136
	Vas	0.172	-1.364	-0.431	0.598	-0.527	-0.167	-	-	-	0.657	-0.445	-0.141	0.321	-0.992	-0.314
**CD34**	Avas	0.193	-1.302	-0.412	0.151	1.437	0.454	0.093	1.681	0.532	0.911	0.112	0.035	0.699	-0.387	-0.122
	**Vas**	0.051	1.955	0.618	0.280	1.080	0.342	-	-	-	***0.005***[**]	***2.800***	***0.885***	0.100	1.644	0.520
**CD39**	Avas	0.764	0.301	0.095	0.151	1.437	0.454	0.313	1.008	0.319	0.575	-0.560	-0.177	0.699	-0.387	-0.122
	**Vas**	0.328	0.977	0.309	0.202	1.277	0.404	-	-	-	***0.049***[*]	***1.970***	***0.623***	0.681	0.411	0.130
CD44	Avas	0.836	0.208	0.066	0.487	-0.695	-0.220	0.642	0.464	0.147	0.416	-0.813	-0.257	1.000	0.000	0.000
	Vas	0.096	-1.665	-0.527	0.911	-0.111	-0.035	-	-	-	0.196	-1.293	-0.409	0.162	-1.399	-0.442
CD45	Avas	0.193	-1.302	-0.412	0.292	-1.054	-0.333	0.911	-0.112	-0.035	0.433	0.784	0.248	0.846	0.194	0.061
	Vas	0.696	-0.391	-0.124	0.623	0.491	0.155	-	-	-	0.604	0.518	0.164	0.150	1.439	0.455
**CD49b**	**Avas**	0.841	-0.201	-0.064	***0.009***[**]	***2.595***	***0.821***	***0.018******	***2.360***	***0.746***	0.911	0.112	0.035	0.627	-0.486	-0.154
	Vas	0.241	-1.173	-0.371	0.280	-1.080	-0.342	-	-	-	0.756	0.311	0.098	0.537	-0.617	-0.195
CD49c	Avas	0.764	-0.301	-0.095	0.924	0.096	0.030	0.218	1.232	0.390	0.575	0.560	0.177	0.561	-0.581	-0.184
	Vas	0.079	-1.759	-0.556	0.377	-0.884	-0.280	-	-	-	0.756	0.311	0.098	0.150	-1.439	-0.455
CD73	Avas	1.000	0.000	0.000	0.501	0.673	0.213	0.312	1.012	0.320	0.822	-0.225	-0.071	0.207	-1.263	-0.399
	Vas	0.621	-0.494	-0.156	0.691	0.397	0.126	-	-	-	0.753	0.314	0.099	0.533	-0.623	-0.197
CD90	Avas	0.686	0.405	0.128	0.439	-0.774	-0.245	0.910	0.113	0.036	0.734	-0.339	-0.107	0.078	1.761	0.557
	Vas	0.060	-1.879	-0.594	0.375	-0.888	-0.281	-	-	-	0.558	-0.585	-0.185	0.063	-1.857	-0.587
CD105	Avas	0.269	1.105	0.349	0.337	0.961	0.304	0.500	0.674	0.213	0.911	0.112	0.035	0.771	-0.291	-0.092
	Vas	0.624	0.490	0.155	0.554	0.591	0.187	-	-	-	0.119	1.560	0.493	0.837	0.206	0.065
CD151	Avas	0.593	-0.535	-0.169	0.260	-1.127	-0.356	0.720	0.359	0.114	0.402	-0.838	-0.265	0.255	1.139	0.360
	Vas	0.396	-0.849	-0.268	0.670	0.426	0.135	-	-	-	1.000	0.000	0.000	0.824	0.223	0.071
CD166	Avas	0.193	-1.302	-0.412	0.292	-1.054	-0.333	0.575	0.560	0.177	0.313	-1.008	-0.319	0.846	-0.194	-0.061
	Vas	0.051	-1.955	-0.618	0.623	-0.491	-0.155	-	-	-	0.756	-0.311	-0.098	0.064	-1.850	-0.585
CD271	Avas	0.920	-0.100	-0.032	0.774	-0.287	-0.091	0.433	-0.784	-0.248	0.313	1.008	0.319	0.081	1.743	0.551
	Vas	0.171	1.368	0.433	0.202	1.277	0.404	-	-	-	0.468	-0.726	-0.230	0.150	1.439	0.455
**HLADR**	**Avas**	0.367	0.902	0.285	***0.028***[*]	***2.203***	***0.697***	0.911	0.112	0.035	0.575	0.560	0.177	0.561	-0.581	-0.184
	Vas	0.241	1.173	0.371	0.377	0.884	0.280	-	-	-	0.254	1.141	0.361	0.150	1.439	0.455

Table 1: Jonckheere–Terpstra test, avascular region was marked with grey background, vascular region was marked with white background. *: p<0.05, **: p<0.01, the significant values were highlighted in bold and italics

Supplementary table 2: Analysing interrater/intrarater reliability and agreement

	ICC		Accuracy	
**Marker**	**inter**	**intra**	**inter**	**intra**
CD34	0.92	0.95	1.63	1.33
CD39	0.58	0.73	45.3	35.97
CD44	0.57	0.38	0.36	0.43
CD49c	1	1	0.85	0.62
CD166	0.94	0.92	8.68	9.96
CD271	0.98	0.99	4.68	3.25

ICC: Intraclass correlation coefficients
